# Invasive Water Hyacinth: Ecology, Impacts and Prospects for the Rural Economy

**DOI:** 10.3390/plants10081613

**Published:** 2021-08-06

**Authors:** Irina Harun, Hafizah Pushiri, Ahmad Juhari Amirul-Aiman, Zufarzaana Zulkeflee

**Affiliations:** Department of Environment, Faculty of Forestry and Environment, Universiti Putra Malaysia, Serdang 43400, Selangor, Malaysia; irina@upm.edu.my (I.H.); noorfizah@upm.edu.my (H.P.); amirulaimanahmad@upm.edu.my (A.J.A.-A.)

**Keywords:** *Eichhornia crassipes*, circular economy, community empowerment, community resilience, green nudge, invasion control, pro-environmental behaviour, waste management

## Abstract

Water hyacinth (WH) is notorious for causing severe environmental degradation and being an economic burden to manage. However, it offers substantial prospects if exploited, especially by rural communities. High temperatures, eutrophic conditions and other environmental factors promote the proliferation of the plant in regions where it has been introduced. Regarded as among the world’s worst invasive weeds, WH is nearly impossible to control and eradicate without an integrated approach and community participation. The effectiveness of control methods varies, yet sustained community involvement determines the long-term success of these methods. Reproducing rapidly, WH has the resource capacity to support a unique microeconomic ecosystem, incentivising WH control by generating sustainable income. The WH ecology, the socioeconomic impacts of its invasion and its various applications are reviewed, and revenue generation and cost-saving options are highlighted. A circular microeconomic model is proposed by integrating WH valorisation into the general limitations of a rural community. Empowering locals with opportunities and enticing them with potential economic gains can be a nudge towards a pro-environment behavioural change in managing WH. This would aid in upgrading local livelihoods and could foster resilience within the community in tackling both environmental problems and economic setbacks through the management of WH invasions.

## 1. Introduction

Invasive in nature, water hyacinth has been extensively addressed in reviews due to its destructive environmental and economic impact [1,2]. Originating from the Amazon, this notorious macrophyte has spread to many other tropical and sub-tropical regions [2,3], invading freshwater waterways, displacing native species, reducing biodiversity [4,5] and deteriorating water quality [6]. In terms of its direct impact on mankind, it disrupts human activities [7,8], acts as a breeding ground for disease vectors [6,9] and continues being a pest in the aquatic environment. Further environmental problems related to water hyacinth are evident. Managing water hyacinth through physical, mechanical, chemical, and biological means causes additional complications [10]. Herbicides used in the chemical cleansing of water hyacinth will pollute water bodies and may bioconcentrate, bioaccumulate and biomagnify in the aquatic food web, potentially eliminating non-target organisms [2]. Introducing a predator, the weevil beetle, for the weed to gain biological control might lead to a secondary catastrophic impact, besides the long duration required to achieve significant success [10]. While they remain the most practical means, physical and mechanical interventions are both costly and labour-intensive [11]. Moreover, once harvested, water hyacinth biomass can create waste issues if not properly managed.

Conversely, a remarkable number of studies have emerged for the potential use and conversion of water hyacinth into value-added products, suggesting a positive aspect of the weed [12,13,14,15,16]. Transforming management issues into opportunities and harvesting water hyacinth through physical and mechanical means by collecting its biomass can be manageable, feasible, and profitable.

Current and recent reviews addressing the valorisation of water hyacinth have focused on several aspects, including water hyacinth biomass-to-energy [15], biochar production potential [12], phytoremediation capacities [17], cost-benefit analyses and the economic feasibility of its utilisation [12,13,18] and various other products [16]. Though frequently mentioned in previous works of literature, the direct and indirect prospects for the utilisation of water hyacinth in affected, and especially rural areas, for the benefit of the people within those areas, has not yet been thoroughly discussed. Most of the literature has focused on the socio-economic impacts that water hyacinth infestations have on the community, rather than on the ways the community can gain immediate benefits from it [7,9,19,20].

Listed among the top 100 in the Global Invasive Species Database by the Invasive Species Specialist Group (ISSG), water hyacinth has been described by many experts as nearly impossible to eradicate. However, the key to its successful control, it has been reported, is the active participation of relevant stakeholders, including the affected locals [21]. However, a lack of enthusiastic involvement in many instances has led to failures and the re-establishment of the aquatic weed invasion [7,22]. Furthermore, the involvement of locals in water hyacinth management has remained limited to roles at the ground level, for example, monitoring the effect of control strategies implemented by relevant authorities or becoming directly involved in the manual clean-up of water hyacinth [21]. Community empowerment to instil knowledge regarding the weed and the prospects of managing it remains deficient.

Promoting pro-environmental behaviours among community members can be vital in addressing environmental problems [23]. One strategy to achieve this is through the introduction of green nudges. Green nudges, or environmental nudges, are informal suggestions aimed at creating a behavioural change, leading to more pro-environmental actions [23,24]. As such, the eradication of water hyacinth and the proper management of its harvested biomass waste could be made economically attractive to rural people through green nudges. This would subtly encourage community members to become actively involved and opt for a more ecological form of water hyacinth management. Some regard nudges as unethical, as they serve to benefit others but overlook the normative cost of the well-being of those being nudged [24]. However, it is often agreed that optimal environmental nudges serve the common good and collective welfare, for both the present and future generations [25]. Therefore, empowering rural communities with the prospects of water hyacinth would not only benefit the environment by making it cleaner, but also the well-being of the locals by potentially allowing income generation. Hence, the normative cost associated with the alleged unethical aspects of green nudging would simultaneously be addressed.

In general, this review will discuss the water hyacinth ecology and the impacts of its invasion on rural communities. Furthermore, it aims to suggest a green nudge: a microeconomic model based on various potential water hyacinth valorisation schemes for the affected rural communities. This would promote the sustainable management of water hyacinth in order to control the expansion of its negative human and environmental impacts. Analysis of the prospects for water hyacinth will focus on the conversion of the weed into two types of products. The first are those that can be created and sold directly by the rural community for income generation and cost-saving purposes. The second type use water hyacinth as their base material and can be supplied by the community to relevant industries. Closing the water hyacinth movement loop in the microeconomic model will also be explored as part of the effort to achieve a circular microeconomy. Rural communities affected by water hyacinth infestation can become involved by coping with and managing the issue through the microeconomic model, which will foster resilience and lead to more sustainable solutions for the water hyacinth control and management issues. 

## 2. Water Hyacinth Invasion 

Water hyacinth is native to the Amazon in South America and especially to Brazil and Argentina. Initially intended to be given as gifts, it was introduced worldwide and has spread both accidentally and deliberately into the natural environment. Invasions have been reported in Africa, Asia, Europe, Central America, North America and the Caribbean [26]. Successful weed invasions are due to the optimum conditions provided by the invaded areas, especially in terms of temperature and nutrient levels, among other relevant factors. According to Wilson et al. [27], at a constant temperature and nutrient level, the projected growth of water hyacinth is a rate of 0.1 kg/m^2^, while under nutrient-rich or eutrophic conditions the rate will increase to 10 kg/m^2^. Supplemented with an average optimum temperature of 30 °C, it takes around 50 days for the plant to reach the 10 kg/m^2^ rate. For these reasons, water hyacinth invasions have been observed to predominantly affect equatorial regions that have warm average temperatures and eutrophic lakes, with rivers and wetlands affected more commonly and severely.

### 2.1. Ecology of Water Hyacinth

Water hyacinth, or *Eichhornia crassipes* (Mart.) Solms, from the family *Pontederiaceae*, is a free-floating aquatic plant that commonly grows in inland freshwater bodies such as lakes, rivers, streams, ponds and wetlands. The plant has broad, wide canopy-like waxy leaves and purple clustered flowers that grow in spikes. The petioles of the plant appear bulbous with air-sacs that help make it buoyant. The plant varies in height from a few centimetres to nearly a metre, while the leaves may be around 15–20 cm in length and width [14]. The plant can sometimes become rooted when it lodges in muddy, shallow waters and the flowers may be blue or white [14]. With the ability to reproduce both sexually through seed propagation and asexually through stolon vegetative reproduction, water hyacinth exhibits the reproductive characteristics suited to invasive success. According to Zhang et al. [26], the invasive spread of water hyacinth is characterised by its genetic uniformity due to its prolific clonal reproduction, dominated through vegetative propagations. In the absence of interspecific competition, water hyacinth outcompetes other aquatic plants, even outside its native range; thus, its growth is rapid and unchecked. Its seeds can remain viable for up to 20 years and may germinate in moist soil or warm, shallow waters [28]. Additionally, the high dispersal of its buoyant propagules and well-developed phenotypic plasticity and its ability to change in response to different stimuli are among the factors that enhance the plant’s adaptive capacity to any local environmental changes in its native range. Meanwhile, these factors facilitate its ability to colonise its introduced ranges [26].

A study by Wu and Ding [5] discussed the abiotic factors in terms of environmental parameters and the biotic, species-specific factors influencing water hyacinth invasion. Interesting highlights from the abiotic study include the influence that the dissolved oxygen level has on reducing the plant growth, while high levels of conductivity, indicating nutrient availability, promotes the plant’s propagation. Water hyacinth has also been proven to reduce the biodiversity of the invaded area, according to four biodiversity indices. Water hyacinth invasion was observed to reduce the overall biodiversity in terms of species richness and evenness. Various other factors may either promote and accelerate or limit and slow a water hyacinth infestation. These include temperature, nutrients, salinity, light, wind, water currents, carbon dioxide levels, waves, turbidity and changes in water levels [27,29]. In general, higher temperatures and greater nitrogen and phosphorus content are important factors promoting water hyacinth growth [27,29]. Higher salinity levels inhibit water hyacinth proliferation by inducing growth reduction and an increase in its mortality rate [30], which hamper invasions in coastal areas. In cases where both salinity and nutrients vary, nutrients will have a greater influence on the leaf count while total biomass remains limited [30]. A higher flow velocity reduces the probability that water hyacinth can become established. Consequently, a higher level of dissolved oxygen derived from the turbulence similarly limits the plant’s growth. Water hyacinth has also been reported to reduce phytoplankton productivities due to the fall in dissolved oxygen and chlorophyll-a levels in water bodies covered by the plant. These have major ecological impacts on the infested water as the aquatic food web becomes disrupted, leading to a drop in aquatic species composition and biodiversity [31].

Despite the extensive knowledge of the inhibiting factors of the plant, the water hyacinth invasion rate can be uncontrollable, especially when aided by continuous inputs of nutrient pollution from agricultural activities. The mechanical harvesting of water hyacinth requires a weed harvester, an excavator and other heavy machinery. Physical harvesting may refer to the same process or involve manual harvesting. Both the mechanical and physical methods lead to an abundance of harvested biomass that must be managed. In contrast, chemical and biological methods aim to eradicate the weed. Chemical cleansing using herbicides kills the plant but may also affect non-target species [2]. Biological control involves the use of biocides or, more commonly, an insect predator of the plant, namely the weevil beetle. Biocides are not commonly used as they are less commercially available compared to their chemical counterparts [32]. Biological control using the weevil relies on its herbivorous nature to shorten the plant petioles and reduce the above- and below-surface biomass [33]. Although successful in reducing the size and increasing the mortality of the plant, weevil biocontrol fails in reducing the plant cover, one of the most important factors in deterring the invasion [33]. With such different approaches and varying levels of success, controlling water hyacinth invasions clearly requires an integrated approach using all available means to ensure long-term success and to avoid the re-establishment of an aquatic weed invasion.

### 2.2. Impacts on Rural Communities

The invasion of water hyacinth has a major impact on the rural people affected, especially those who depend on water bodies for their livelihoods, such as fishing and riparian communities [7,8,9,20,34]. As discussed by Dersseh et al. [1], a water hyacinth invasion has negative impacts on the hydrology and environment, resulting in subsequent socio-economic impacts, as it disrupts human daily activities and health. The increase in evapotranspiration compared to surface evaporation disrupts the hydrological water balance in the infected areas, which could disrupt local rainfall events. Reduced water flows in rivers due to water hyacinth blockages will promote sedimentation, deoxygenation and water quality deterioration. Weed canopies on lakes reduce sunlight penetration. This increases the water turbidity and reduces variability in temperatures, as well as other similar water quality concerns [35]. Consequently, all these events lead to a reduction in fish and other aquatic organism populations as their habitat becomes less habitable. Instead, the proliferation of disease vectors such as mosquitoes and snails will occur, as the plant hosts a variety of these species [9].

Dense weed mats mean limited access to waterways, leading to conflicts among the affected communities to gain access to watercourses. As water hyacinth is buoyant and not anchored, it moves with the wind. This is especially disruptive to fishermen, making boat navigation harder, delaying fishing preparation and resulting in fishing net entanglements and damage to other equipment [20]. In places with hydroelectric dams, the invasion has led to damaged generators and coolers and threats to the electricity supply [28,36]. Hence, locals’ livelihoods are disrupted as many lose their source of income, incur costs due to damage and are further inconvenienced in many ways.

Coping strategies for water hyacinth invasion can be described as reactive- or recovery-based [34]. Reactive behaviour is a short-term coping mechanism for handling an immediate current situation. Affected communities tend to be reactive, for instance, by joining clean-up activities to remove the weed or simply halting their daily routine during the invasion peak. On the other hand, some communities have been reported to adopt alternative sources of income when interrupted by water hyacinth. Such actions are considered recovery-based, as locals recover their livelihoods through other means, such as switching to agriculture.

From the rural perspective, water hyacinth has only negative impacts on communities, according to a survey conducted on communities affected by an invasion of Lake Tana, Ethiopia [7]. Livelihood security concerns may hinder the prospects for water hyacinth to be explored by a rural community, especially without support from relevant authorities, which can provide information and technical and financial aid [37]. Thus, a community tends to adopt alternatives to overcome its hardship. Consequently, the water hyacinth problem remains unresolved. 

Therefore, a community must be empowered so that it becomes resilient in coping with a water hyacinth invasion. The problem tends to recur because fully eradicating the plant appears to be impossible as it rapidly expands its territory. The key to successful community involvement is the dissemination of information coupled with empowerment programmes. These approaches would enlighten locals, offering not only the knowledge but also the skills needed to manage water hyacinth for their benefit. One example of a successful empowering project was reported in Indonesia. The Bangkit Bersama Cooperation, a community empowerment institution, developed a water hyacinth waste scavenger programme in the Saguling Reservoir area that created job opportunities through the utilisation of the weed [38,39,40]. The success of such programmes highlights the importance of integrating the concept of the weed into the community and local livelihoods in order to ensure that its management is sustainable [37].

## 3. Prospects for Water Hyacinth 

Despite being one of the top ten global worst weeds, water hyacinth is also considered a highly productive plant [41]. Its rapid reproduction provides numerous opportunities for its usage as a sustainable resource. In that sense, water hyacinth is not merely an environmental challenge, as its considerable inherent advantage can be utilised for economic growth [42]. The valorisation of water hyacinth is not new; its prospects are extensive, encompassing different sectors such as agriculture, energy, metallurgy, construction, pharmacology, arts and craft, material science and more [13,15,16]. Despite these various opportunities, rural communities may not benefit directly from all the applications as limitations may arise due to the location, the cost and the complex technology required and the availability of labour. The following section reviews the prospects for water hyacinth in terms of how it can be managed, reused, recycled or repurposed by rural communities, as well as how it can be merely supplied to other relevant industries by the community. The community’s role within the water hyacinth management cycle for each application is also discussed.

### 3.1. Water Hyacinth for Feeds

Water hyacinth has been studied for its nutritional value and potential use as feed for livestock, poultries, and fish [43,44,45,46]. Its nutritive attributes—for example, it is high in cellulose, hemicellulose and crude protein content—make it suitable for use as a substitute or an additive for animal feeds [46]. 

Water hyacinth has been reported to be used as feed for cattle [47,48], goats [49,50,51,52], sheep [53], pigs [54,55], ducks [56,57], rabbits [58,59], fish [45,60,61,62,63] and other animals.

Water hyacinth is highly versatile: it can be used directly as fresh feed [54,56], ensiled into silage by mixing with manure, urea, molasses, straws and other substances [48,64,65], composted [66], or used in a dried or wilted form as hay [47,53,67] for feeding animals. The plant’s relatively low fat content, however, prevents voluntary uptake by certain livestock as it lowers palatability [59]. Ensilaging is regarded as a more common practice, as it helps to make the water hyacinth feed more palatable to animals [65]. Similarly, wilted water hyacinth was stated to reduce digestibility and intake as feed, yet it was also reported as an economically viable substitute in the areas it invaded, as its availability is guaranteed [46]. Another major disadvantage of using water hyacinth as feed is the high crude fibre content of the plant, which may lead to a lower feed conversion (feed/weight gain) ratio and can be an anti-nutritional factor for certain animals [63]. However, this can be overcome through fermentation with added microbes [57,63].

The positive effects of utilising water hyacinth as feed include increased weight gain in pigs [55], increased feed intake and digestibility of nutrients in bulls [68] and cattle [48], higher final protein content in fish [63] and goat meat [69,70], strengthened duck eggshells [56], improved microscopic aspects of goat sperm [70] and lower feed production costs for rabbits [58,59] and fish [66]. Some negative health effects do arise, especially related to digestion, when ruminants are fed directly or solely with the plant [7,46]. Meanwhile, water hyacinth was also reported to have no additional effect when mixed with other concentrated feed ingredients or used as additives. In fact, an increase in its ratio may have detrimental effects [63,71,72].

Additionally, water hyacinth nutrient contents were proven to be independent of its place of origin and remain constant at the same levels, even if collected from different geographical areas or water sources [44,73]. Although normally the whole plant is used as feed, some users omit the roots to avoid possible metal contamination, even when the metal concentration was found to be below the maximum permissible limit [59]. In general, leaf meal was observed to be more preferable, as the water hyacinth leaf protein content accounts for about 50% (per dry weight) of its total nutritional quality [74].

**Figure 1:**
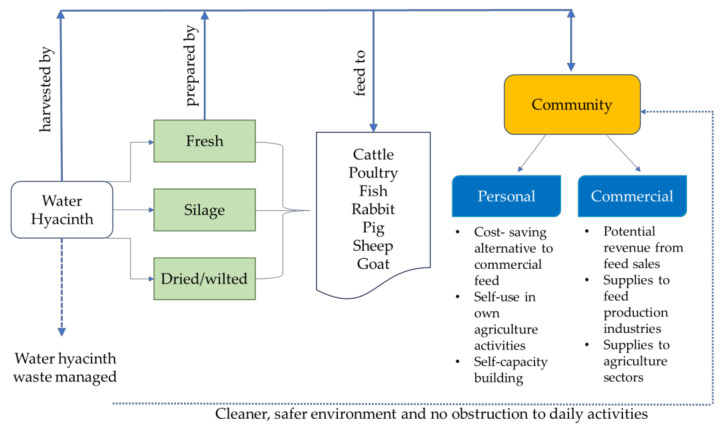
Water hyacinth, its animal feed potential and the benefits that can be gained by the community.

For decades, livestock agricultural activities have played an important role in the rural economy [75]. Promoting suitable incentives for reducing feed costs, for example, by utilising water hyacinth, may improve rural livelihoods that depend on livestock agriculture. With the diverse possibilities of using the plant for animal feed, rural communities affected by water hyacinth invasion can take advantage by utilising it for rearing animals, whether at the personal or commercial level. Water hyacinth application, with no amendment besides sun drying, is suitable for small-scale use and incurs relatively minor nutrient losses for feed. In addition, less of a workload would be required [76]. Using them fresh or dried incurs no costs, although for better results, ensilaging or fermenting the plant for feed can lead to higher animal acceptability and subsequent higher returns. However, the latter might require some form of intervention and involve knowledge transfer programmes delivered by relevant parties to educate the communities on the right methods. This would arguably mean that managing water hyacinth as feed could be practised sustainably. Figure 1 summarises the potential use of water hyacinth as feed and the role of the community in the management cycle of the plant.

### 3.2. Water Hyacinth for Biofertilisers

Harvested water hyacinths left to dry on land can quickly wilt and will, in time, be naturally composted. Cleared land with a pile of water hyacinth leftovers will quickly enter secondary succession driven by the nutrients provided by the composted weed through natural fertilisation. Rich in organic nutrients, water hyacinth consists of more than 70% organic matter on a dry basis [14] and high levels of nitrogen (N), phosphorus (P) and potassium (K) content [13]. Water hyacinth can be either mulched [77,78,79], composted [17,80] vermicomposted [81,82] or anaerobically digested [83] for biofertilisation purposes. 

Mulching involves using the fresh or dried water hyacinth with or without amendments for soil cover to retain moisture, stabilising the soil structure, regulating soil temperatures and controlling the weed [79]. Composting usually involves mixing water hyacinth with animal dung, with cow dung being the most common [17,77,84]. Composting with the aid of worms describes vermicomposting. In this process, water hyacinth supplies nutrients to both the soil and worms and the excreted worm casts further improve the overall soil condition [81]. Anaerobic digestion involves mixing water hyacinths with various wastes, including animal manure, solid waste and food waste for biogas production. The resulting digested liquid and solids can then be used as biofertilisers [83,85].

Mulched water hyacinths have been reported to contain 10–40% more nitrogen (N) and 20–50% more carbon (C) compared to other aquatic weeds, increasing the quality of the biofertiliser [77]. Moreover, digestates from the anaerobic digestion of water hyacinth have been reported to be effective biofertilisers because they contain phytohormones, nutrients (N, P and K) and other bioactive compounds that support plant growth [85,86]. Composting water hyacinth can reduce metal bioavailability in the plant, especially in specimens obtained from polluted waterways or used in phytoremediation [80]. Further metal leachability reduction can be achieved through vermicomposting [84]. These methods of managing water hyacinth ensure the safer application of the composted plant as biofertilisers, especially for edible crops.

In general, biofertilisers made from water hyacinth can improve soil nutrient content, increase crop growth, improve crop quality, and even curb weed and pest infestation in certain cases [78,82]. However, depending on the type of mixture and condition used when preparing water hyacinth biofertilisers and the type of crop it is used on, beneficial outcomes may vary. Water hyacinth has been shown to improve soil nutrient content for strawberry growth, but the strawberries do not benefit directly [87]. When used as mulch, water hyacinth was reported to improve soil temperature, soil moisture and crop yield in maize cultivation [79]. In turmeric farming, water hyacinth mulch improved the morphological and physiological characteristics of the tuber plant [78]. Water hyacinth mulch was also reported to be able to lower the soil salinity in saline soil for the cultivation of potatoes and tomatoes [88], while composted water hyacinth helps overcome salinity stress in white lupin seedlings [89].

**Figure 2:**
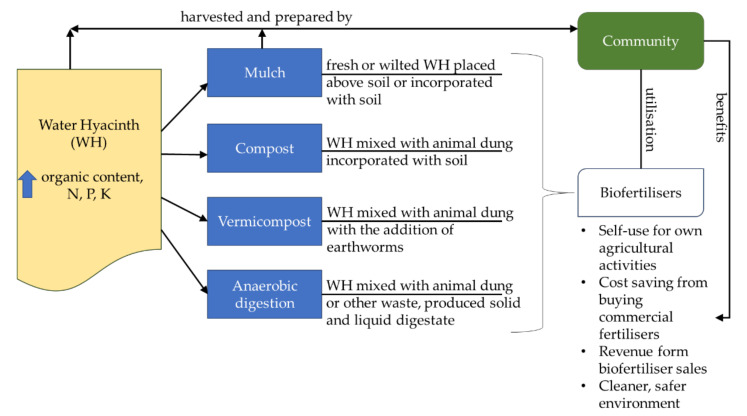
Water hyacinth valorisation to biofertilisers and the role and benefits of the community.

The various benefits for the plants and crop growth indicate that water hyacinth-based biofertilisers should be sufficiently attractive for affected rural communities to take advantage of. Mulching and composting are more feasible than vermicomposting and anaerobic digestion, and little training is needed. Additional community empowerment can facilitate greater awareness among other communities in terms of the prospects for using water hyacinths as biofertilisers, while interest in vermicomposting and anaerobic digestion can be aided by providing training and knowledge transfer. Hence, water hyacinth waste can be properly managed, and enhanced crop production will help improve community livelihoods. A summary of the weed’s biofertilisation potential and its connection with the community is portrayed in Figure 2.

### 3.3. Water Hyacinth in Crafts

Water hyacinth has great potential for use in craft production. Raw material from the dried plant and its fibre can be utilised to make bags, handbags, wallets, flower pots, fashion accessories, mats and many other items [90,91,92]. According to Rakotoarisoa et al. [93], in selecting the raw materials, the water hyacinth stem length must be at least 50 cm for it to be suitable for handicraft production. They also added that water hyacinth stems are simple to cut and weave due to their size and flexibility. Other than the stem, the dried petioles of water hyacinth are also used to make other forms of handicraft, including coasters, mats, shoes, sandals, belts, wallets and vases, in countries like Indonesia and the Philippines [94]. Today, digital technology has enabled the development of home-based enterprises (HBEs) or home industries utilising water hyacinths. For instance, communities around Rawapening in Indonesia utilised overgrowing water hyacinths in online home-based enterprises [95]. The locals use water hyacinths to make products such as bags, sandals and baskets, and furniture like chairs and tables, before marketing them using the internet [95]. In Madagascar, communities traditionally use papyrus for handicraft production; however, water hyacinth is now being used in the production of large and small hats, shopping bags, handbags, sandals and mats [93]. These products are also being exported to international markets [93].

The results of water hyacinth processing helps to empower the community. Usually, handicraft production using water hyacinth involves the selection of raw materials, the transportation and drying process, the ornament choice and, finally, the construction and production of the items [93]. In Indonesia, for instance, the Bangkit Bersama Cooperation has developed an empowerment programme to train people in processing water hyacinths for craft production. Housewives have been taught how to turn water hyacinth weeds into a variety of craft items, including bags, vases, tissue boxes, calligraphy, furniture and many other products [39]. It was found that the resulting art and craft works are enabled by the collaboration between the Bangkit Bersama Cooperation and the community. The products are then sold domestically and internationally. Another community empowerment effort in Indonesia can be identified in a Samosir regency government policy that exploits water hyacinth in Lake Toba for bag-making, a product targeted at elementary to high school students. This benefited the community, especially the unemployed, as new bag-making job opportunities were created. Besides providing the capacity for the community to cope with environmental problems, the method can also reduce the poverty levels associated with the lack of available jobs [96]. The communities were also provided with waste management expertise and skills, ranging from identifying different forms of waste-to-waste separation to conditioning and recycling [39]. 

The involvement of women in industrial crop processing practices can also enhance their ability to contribute significantly to household budgets and decision-making, leading to long-term empowerment. Housewives who traditionally did not work and relied financially on their husbands can now assist their partners by supplementing their income [97]. For instance, many housewives operate small stalls from their homes where they sell regular necessities. Ristianasari et al. [98] mentioned that communities who have had a long-term engagement in empowerment events and are participating in organisations appear to be more independent, and so empowerment projects have been demonstrated to offer knowledge, widen perspectives and inspire people to access the available improvements. Nieminen et al. [99] added that locals’ constructive internal resources were strengthened, their inner mind functions were enhanced and their lives were given greater meaning through such experiences.

**Figure 3:**
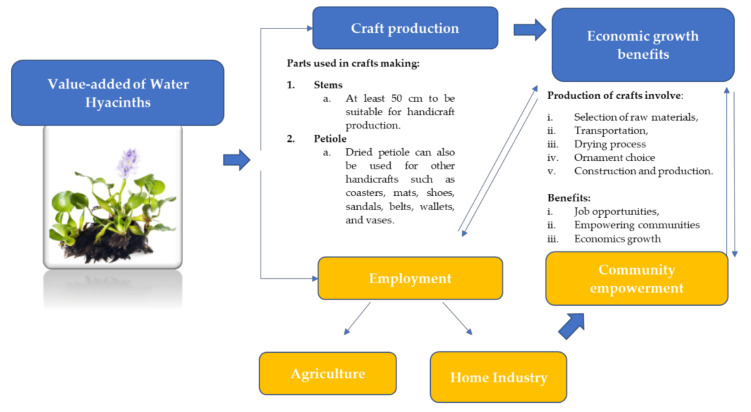
Illustration of value-added water hyacinth to economic growth and community empowerment.

After becoming empowered through a social compass approach, residents’ socio-economic systems in Indonesia, for instance, have shifted from agriculture to home industry. Working as traders or craftsmen allows people to meet their everyday needs and, in conjunction with garbage scavenger and women’s empowerment programmes, creating handicrafts from water hyacinth waste is becoming an appropriate way to integrate economic gains and social empowerment while utilising a plant that has tended to be seen only as a problem [39]. The added value of water hyacinth in terms of economic growth and community empowerment is summarised and shown in Figure 3.

### 3.4. Water Hyacinth Conversion to Bioenergy

Water hyacinth has immense potential for use as an energy resource. Due to its dense population and aggressive growth, the utilisation of this aquatic weed as an energy feedstock is highly attractive, especially when paired with its potential capacity for phytoremediation and energy production. Furthermore, using this biomass as an energy resource solves the issue of water hyacinth management post-phytoremediation [13]. Water hyacinth is abundantly available, biodegradable and characterised as a non-crop plant. This categorises the biofuels thus produced as second-generation biofuels, alleviating any food versus fuel complications. Its biomass characteristics are promising in terms of energy production; it has a low lignin content (10%) and high cellulose (20%) and hemicellulose (33%) content [100]. As the lignin content is low in water hyacinth, the plant is especially suited for utilisation as a bioenergy resource, as this compound hinders the fermentation processes of several commercial yeasts and enzymes [100]. Water hyacinth also exhibits a useful C/N ratio within the range of 20:1–30:1, which is appropriate for microbial decomposition processes [101]. 

**Figure 4:**
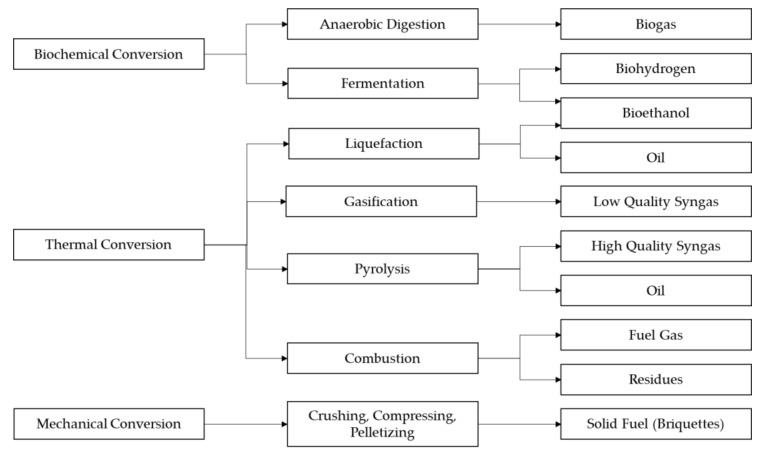
The energy potential of water hyacinth biomass.

These features make it suitable for use as feedstock for many types of biofuels (see Figure 4), including biogas through anaerobic digestion; biohydrogen and bioethanol through hydrolysis and fermentation; syn-gas, biochar and bio-oil through thermochemical conversion via pyrolysis, gasification and hydrothermal liquefaction (HTL); and briquettes through mechanical conversion. However, fresh water hyacinth biomass may contain up to 95.5% water, which may complicate harvesting and processing [100]. The biofuel yields from thermal processes such as combustion, gasification and pyrolysis generally suffer from wet biomass resources, which necessitates pre-treatment and dewatering. As such, the scope of this review is limited to the biochemical and mechanical conversion of water hyacinth biomass via biomethane (biogas) and briquette production. This is based on the intention of the authors to focus on economical nudges for rural communities, which require that approaches are less technologically complex yet economically feasible, as well as being based on locals’ socio-economic capacity and the existing energy situation.

#### 3.4.1. Biogas Production through the Anaerobic Digestion of Water Hyacinth

Anaerobic digestion is a process whereby organic matter is converted into biogas, a mixture of methane (CH_4_) and carbon dioxide (CO_2_). Biomethane can then be used as an energy resource for heating, cooking and power generation. This conversion relies on the biochemical activities of bacteria and archaea consortia that break down the complex organic matter into soluble monomers such as amino acids, fatty acids, simple sugars and glycerols, subsequently producing methane. 

In detail, four stages are involved in the anaerobic digestion process: hydrolysis, acidogenesis, acetogenesis and methanogenesis. Initially, the biomass-containing complex organic compounds undergoes hydrolysis, whereby they are broken down by the consortia into their monomers of simple sugars, amino acids and fatty acids. The hydrolysis of carbohydrates usually concludes within hours, whereas the process for lipids and proteins may take days to complete [102]. The simple monomers will then be fermented in the acidogenesis stage, which produces volatile fatty acids (VFAs), alcohols, hydrogen (H_2_) and CO_2_ [103]. The product in the second phase becomes the feedstock for the bacteria involved in the third acetogenic phase. These microorganisms reduce H_2_, CO_2_, organic acids and alcohols to acetic acid. Then, depending on the methanogen population available, acetate, methanol or H_2_ and CO_2_ are converted into methane in the final stage, the methanogenesis phase. Current AD processes usually occur in a single stage and the biogas yield greatly depends on the population of the microorganisms in the consortia and sufficient mixing for feedstock contact. However, as anaerobic digestion contains four distinct stages, the stability of the methane production process is influenced by the methanogen activity in the consortia. Methanogens have slower growth rates compared to other microbes and thus present a challenge. In their study, Barua and Kalamdhad [104] concluded that a two-stage biogas digester is superior to traditional single-stage digesters, as a higher rate of feedstock degradation, a more stable process and a simpler biogas recovery system were observed. The two stages of the process separate the methanogenesis phase from the other phases and ensure that optimum environmental conditions for the methanogens can be maintained in order to maximise methane production. 

Another bottleneck is the hydrolysis stage, in which the sugar monomer yield becomes the limiting factor in subsequent stages; this eventually determines the biogas yield. However, water hyacinth proved useful by achieving higher reducing sugar yields and faster hydrolysis rates than other aquatic and terrestrial plants such as water peanut, miscanthus and metasequoia chips under the same conditions [105]. This highlights an opportunity to use water hyacinth in anaerobic digestion processes, which could be adopted by communities that are severely affected by the invasion of water hyacinth and economically dependent on agriculture. They would be able to control the weed, generate income and become energy-independent. However, many factors affect the performance of biogas production, including the operating parameters, feed characteristics and mode of operation. These will be reviewed briefly to highlight the links between the availability of water hyacinth as feed, the potential of a circular economy through a zero-waste philosophy and the requirement of simple technologies that rural areas can implement easily. 

Operating temperature is a highly significant parameter for anaerobic digestion. There are three temperature profiles, psychrophilic (T < 20 °C), mesophilic (20 °C < T < 40 °C) and thermophilic (T > 45 °C), although anaerobic digestion processes usually occur in either mesophilic or thermophilic mode [106]. Mesophilic reactions are primarily used as thermophilic digesters that require higher energy input and are sensitive to operational parameter changes [107]. From the rural community perspective, therefore, mesophilic AD offers an obvious advantage compared to the thermophilic process, as it requires very minimal energy input and is less complicated. However, Photong and Wongthanate [108] demonstrated that cumulative methane production is higher when the process occurs at a thermophilic temperature of 55 °C (230 mL/g_COD_ methane produced), compared to a mesophilic temperature (methane production at 110 mL/g_COD_). Methane yield rises at higher temperatures have been linked to lower enzyme activation energy, higher hydrogen production, stable consortia population and increased methane cumulative production [109,110,111]. Overall, many processes involved in anaerobic digestion are biochemical and highly dependent on the reaction rates of the enzymes involved [106]. The characteristics and optimal conditions of these enzymes, however, depend on the microorganism used in the AD process.

The existing microbial community in a digester is highly dependent on the chemical and physical characteristics of the feed and the initial inoculum [112]. Common forms of inoculum used in the AD process include farm animal manure, municipal sewage sludge and leachate from composting plants [113,114]. For example, the addition of rumen fluid in the range of 25–50% during the pre-treatment process showed optimum biogas production compared to runs without this addition [15]. Inoculum starter is a prerequisite at the commencement of the digestion process; however, subsequent processes may occur without the addition of inoculum. Nevertheless, the absence of inoculum causes more dynamic variations in the microbial community, leading to increased lag time and lower biomethane concentration, regardless of pre-treatment [111]. From the perspective of rural communities planning to adopt the AD approach to treat their agricultural or municipal waste, the use of inoculum is an interesting opportunity, as locals could use any available inoculum source from their economic activities, such as waste from cow-herding, poultry farming or fishing. 

The operation of anaerobic digestion can proceed in either liquid or solid-state, which is determined by the total solids (TS) content. For liquid AD, TS (% *v*/*v*) values range between 0.5–15%, while solid-state AD has values higher than 15% [115]. AD processes may also occur with single feed or feeds co-digested with other substrates, such as food, agricultural or animal waste [114,116,117]. For example, Photong and Wongthanate [101] studied the feasibility of using a mix of water hyacinth and cassava starch sediment for biomethane production. They discovered a ratio of water hyacinth and cassava starch sediment at 25:75, initial pH of 7.5, thermophilic temperature (55 ± 2 °C) and C/N ratio of 30 were optimal for the digestion process and produced 436.82 mL CH_4_/g_COD_. One advantage of co-digesting water hyacinth with other substrates is that the stability of the process can be improved. The addition of water hyacinth to food waste in the digester feed was found to maintain pH values in the optimum range as the plant acts as an organic nutrient and buffer agent [118]. Zhao et al. [119] also argued that the co-digestion of food waste and waste-activated sludge mitigates the effects of salt and pH variation on biogas production. After all, pH is another factor that affects AD performance. In general, the anaerobic digestion process is conducted within the neutral pH range of 6.6 to 7.8, as lower pH values tend to modify the biochemical activities of the methanogen enzymes into non-methane metabolites [107]. The presence of salt in the feedstock is also a point of contention as it has been found that high concentrations of chloride salts (4–10 g/L) in digester feed inhibits biomethane production as methanogen growth is affected [110]. However, in some cases, a higher salt concentration does not delay biogas production but, instead, promotes hydrolysis and the acidification steps of anaerobic digestion through the promotion of the hydrolysis enzyme activity. This maintains the biofilm balance and regulates the osmotic pressure in the cells [119]. Methane production in the digestion of water hyacinth from brackish water (22.5 L/kg VS_added_/day) was twice the rate of that from freshwater (10.0 L/kg VS_added_/day) due to the higher nutrient content in the former [120]. In conclusion, an optimum level of salt is required to maintain the optimum conditions in the digester. 

With regards to the feed, it has been suggested that methane yield can be improved by employing pre-treatment to the water hyacinth biomass. Four approaches can be applied [106,113,121]: (i) mechanical pre-treatment in which the solid sizes are reduced through shredding or grinding, which assists hydrolysis by making more surface accessible to enzymes; (ii) thermal pre-treatment, which uses high temperatures to assist the physical degradation of biomass; (iii) chemical pre-treatment, which uses alkali or acid to improve the carbohydrate fibre digestibility; and (iv) biological pre-treatment, which uses external enzymes or microorganisms to convert complex sugars into monosaccharides. These approaches are readily available to communities and would improve the characteristics of the water hyacinth available before it is used in the subsequent processes and transferred to the AD system. Nevertheless, for AD purposes, mechanical and thermal pre-treatments are relatively easy to apply and require only basic machines or items, which are likely to increase their appeal among rural communities. Despite the advantages of high-temperature pre-treatment processes, low temperatures can be utilised to further simplify the process. However, the addition of alkali is required, as noted by Carlini et al. [106] Their paper also demonstrated that through this approach, the reduced sugar concentration increased from 57% to 74% (*w*/*w*), while the ash content decreased. A multiple pre-treatment approach might also increase biomethane production. Xu [122] concluded that reducing the water hyacinth biomass size, coupled with thermophilic conditions, improved biomass biodegradation by up to 59%.

The energy potential of water hyacinth is substantial and encouraging. Castro and Agblevor [118] estimated that 846.5 MJ of energy can be produced from one tonne of fresh biomass and only 6.8% of that energy produced is required for mechanical harvesting. Therefore, although energy is required to harvest the water hyacinth biomass from its aquatic environment, the total energy produced from the biomass is more than sufficient to maintain continuous operations. In terms of productivity, 50 kg/m^2^ of ash-free water hyacinth biomass can be produced annually with a daily biomass productivity of 0.04–0.08 kg/m^2^ per day [93]. From the harvested biomass, the annual energy potential from one tonne of biomass is equivalent to 13.3 m^3^ biogas or 18.35–18.75 kWh electricity [108]. If a higher methane concentration of up to 75% is achieved, the electricity output may increase up to 25 MJ/kg [116].

#### 3.4.2. Briquette Production from Water Hyacinth Biomass

Despite its substantial potential as an energy resource, the direct utilisation of water hyacinth is relatively challenging. Fresh biomass is bulky and has a low density, so its use in conventional burners is uneconomical. Briquetting is a biomass densification process that transforms biomass residues into a cleaner and enhanced solid fuel with higher density and heat intensity (13.1–18.4 MJ/kg) [15]. Raw water hyacinth contains up to 95.5% moisture, which compromises its ability to be combusted as a direct fuel [14]. The advantage of converting biomass into briquettes is that the moisture content can be reduced while its density is increased, which enhances its fuel properties [100]. The transformation of water hyacinth biomass into briquettes is also a more environmentally friendly process, as co-firing with coal reduces greenhouse gas emissions [123]. 

The fuel properties of briquettes made from water hyacinth also depend on the biomass ratio, which subsequently determines the briquette’s physical, mechanical, and thermal properties [124]. Photong and Wongthanate [108] demonstrated that ratios of water hyacinth (WH) and cassava starch (CS) sediment at 10:90, 20:80, 30:70, 40:60 and 50:50 were optimal as fuel properties, with heating values of 15.66, 15.43, 15.10, 14.88 and 14.58 MJ/kg, respectively. Previously, many different types of agricultural waste had been tested in briquette production using water hyacinth. These included waste fibres from the oil palm industry, sawdust, tannery solid waste and fruit waste [124]. 

The most important factors in bio-briquette production are moisture, cellulose and lignin content [108]. Lignin enhances the bio-briquette binding characteristics while low moisture content ensures that high-quality briquettes are produced by avoiding instances where incomplete burning and fly ash formation are detected. Cellulose and lignin ratios also influence the volatile matter content, which subsequently affects the combustion rate and calorific value of the briquettes. For comparison, briquettes with a volatile matter content in the range of 65–85% were shown to coincide with heating values in the range of 12.91–16.00 MJ/kg. 

In the briquetting process, the raw materials are processed as follows: first, the raw biomass is ground into a uniform size suitable for the subsequent processes. Then, they are dried to significantly reduce the water content. When the optimal water content has been achieved, a briquetting machine is used to form the biomass into briquettes. This densifying process functions due to the binding properties of lignin at a high pressure and temperature, which makes the various biomass components adhere together. However, studies have demonstrated that low pressures may also be used to achieve comparable results. This may appeal to rural communities, as relatively simple technology can therefore be used and only hand-pressed mechanical equipment is required [124,125].

**Figure 5:**
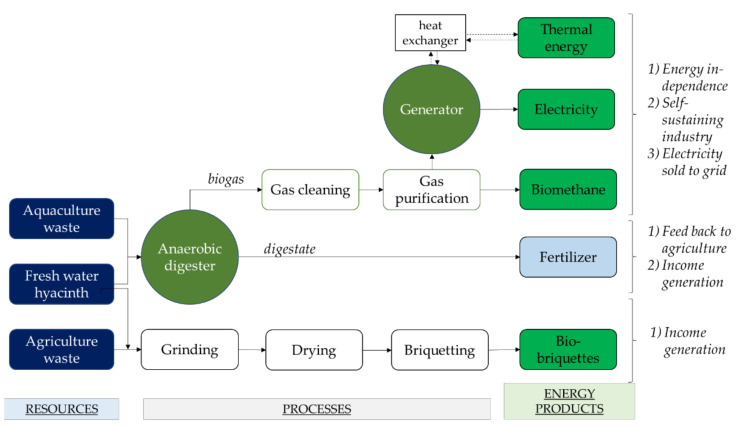
Bioenergy production for uptake by rural communities.

Implementing both AD and briquetting processes for bioenergy production in rural areas is a holistic approach to treat WH. Since rural communities tend to rely on economic activities such as agriculture, farming and fishing, many potential co-digestion and co-briquetting candidates are available for the anaerobic digestion and bio-briquette process. The manufacture of products such as briquettes, which can be marketed, and biomethane, which can be used locally to provide power, is a nudge from which rural communities can benefit (Figure 5). Small and independent industries can utilise the new energy resources to cut their energy expenditure, while utilising the available waste streams means that no additional waste management costs are incurred. This allows communities to sustain their small industries, while local jobs are created, eventually leading to wealth creation. In addition, the environment also benefits from such endeavours as the available waste from the associated industries, which are treated systematically, reducing carbon emissions and pollution. 

### 3.5. Supplying Water Hyacinth to Other Industries

According to some reports, water hyacinth has been used as a raw material for bio-based building materials such as thermal insulators [126,127] and concrete mixture [128,129]. It has also been used in the production of high-value chemicals such as furfurals and hydroxymethylfurfural (HMF), biopolymers and enzymes, as reviewed by Ilo et al. [13] and the production of biochars used in agriculture and low-grade energy sectors [130,131]. Water hyacinth has other potential bioenergy uses, having been extracted for its phenalenone compounds and sterols for pharmacological purposes [132] and used for water treatment purposes through phytoremediation [133].

As water hyacinth is commonly grown and proliferates in slightly polluted to polluted environments, its phytoremediating potential has been investigated more often than its other recognised prospects, which can be considered newer breakthroughs. The following section focuses on the prospects for the use of water hyacinth in phytoremediation and the link to the metal reclamation industries that might be supplied by the affected rural communities.

#### Phytoremediation and Metal Reclamation

Water hyacinth has a proven capacity to remove heavy metals from wastewater [134,135,136,137]. A study reported that water hyacinth can remove up to 99.5% of chromium (Cr(VI)) pollution from industrial mine wastewater [134]. It was also reported that water hyacinth was able to remove zinc (Zn), lead (Pb), iron (Fe), copper (Cu) and nickel (Ni) from landfill leachates [135]. Besides heavy metals, water hyacinth has a reported capacity to remove ammonia (NH_3_) and phosphorus (P) from sago mill effluent [136]. The removal of P by water hyacinth from rice mill wastewater was reported to be up to 77.2% efficient [137].

Water hyacinth removes heavy metals by root uptake and accumulates them in the plant. While some heavy metals are transported to other aerial parts of the plant via the xylem, the root accumulates the majority of the contaminants, possibly in multifaceted vacuoles in the root tissues [135]. Considering the high accumulation of heavy metals in the root and the direct exposure of the root to the wastewater heavy metals, it is understandable that the root suffers the greatest stress due to the toxicity of the heavy metals, causing it to be thin and wiry, as observed by Abbas et al. [135] Additionally, while the plant growth rate was positive when grown in wastewater [137], it was negatively affected compared to its growth in clean tap water and can be identified by the stunting, chlorosis, and shorter root length [135]. However, despite the negative effects of the heavy metals on plant growth, it was reported that heavy metal removal by water hyacinth peaked at 50–75% wastewater dilution [135,137].

The ability of water hyacinth to remove heavy metals from wastewater creates a valuable opportunity for its direct application in the wastewater treatment process. Unlike physiochemical wastewater treatment methods, the application of water hyacinth in wastewater treatment does not require the utilisation of energy, chemicals or the production of adsorption materials. Additionally, the accumulation of heavy metals in water hyacinth allows contaminants to be easily removed. Used and collected water hyacinth can be composted, the heavy metals from which can be recovered using electrocoagulation, electro-floatation or a combination of the two [138]. The recovered metals can be formed into solid bars and sold to metallurgical industries, like the process flow adopted by e-waste recycling facilities in Malaysia [139].

**Figure 6:**
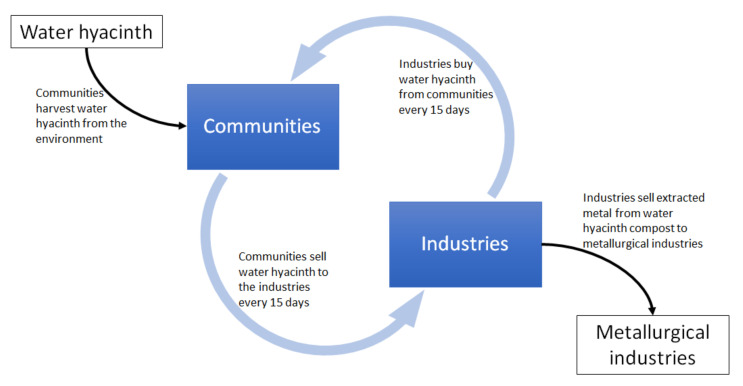
The water hyacinth supply and demand cycle when used as an industrial wastewater treatment. The plants will be extracted by the communities and sold to the industries. The latter use the whole plant to collect heavy metals from their wastewater. The industries can then compost the whole plants before extracting the heavy metals from the compost and selling them to metallurgical industries. Since water hyacinth can significantly reduce heavy metal levels in wastewater within 15 days, industries will be prompted to buy more water hyacinth from the communities after this period to treat the next wastewater batch. This cycle demonstrates how communities will benefit from the sale of water hyacinth and the industries will benefit from the cheaper wastewater treatment and sale of extracted metals from the water hyacinth compost.

The phytoremediation potential of water hyacinth in removing heavy metals contamination from wastewater treatment reveals an opportunity for communities with water hyacinth infestation problems. The current practice of removing the infestation with no monetary returns can strain communities’ livelihoods. Instead, the unwanted water hyacinth can be harvested and sold to industries for use in their wastewater treatment process. Moreover, the direct application of water hyacinth in wastewater treatment allows plants to be sold immediately and with no further processing, making the process sufficiently simple to be performed by these communities. Furthermore, significant heavy metal removal by water hyacinth can be achieved in 15 days [135,137], which would prompt industries to buy more water hyacinth periodically from the communities. The constant industrial wastewater production means a constant demand for fresh water hyacinth supply from the communities. This continuous water hyacinth supply and demand would benefit three parties: (1) the environment (due to the removal of water hyacinth, a pest, from lakes); (2) the communities (healthy lakes allow fishing; the sale of water hyacinth provides profits); and (3) the industries (due to the cheaper wastewater treatment system and profits from heavy metals extraction) (Figure 6).

## 4. Microeconomics of Water Hyacinth for the Rural Community

Experiencing environmental disturbances such as a water hyacinth invasion jeopardises not only the livelihoods of rural communities, but also their health and the environment. Water hyacinth invasion tends to be long-lasting without continuous, integrated management from all stakeholders. Wainger et al. [140] proved that the benefits of managing water hyacinth outweigh the long-term costs of controlling it.

Hence, it is imperative that the community becomes resilient in coping with the problems arising from an invasion. Some core elements of community resilience are local knowledge, health, resources, the interconnectedness of the community through networks and relationships, communications, economic growth and preparedness, among others [141]. According to Rijke et al. [142], the adaptive capacity of rural communities in becoming more resilient to environmental disturbances can be supported by circular economy approaches in resource use that are available within the community. The continuous growth dynamics of water hyacinth ensure a sustainable supply of the weed as resources for various valorisation strategies [3]. 

Therefore, to gain enthusiastic commitment from rural communities affected by water hyacinth invasion, a microeconomic model based on water hyacinth valorisation is suggested, using a circular economy approach. This model aims to be a nudge towards a pro-environmental behavioural change within the community in terms of their handling of problems related to water hyacinth. An effective environmental nudge would help attain environmental goals, ensuring that the benefits outweigh the costs and remain ethically sustainable in the long term [25]. With a clear environmental aim that focuses on a community’s potential gains, it is believed that the circular micro-economic model can be a useful environmental nudge towards sustainable water hyacinth management.

**Figure 7:**
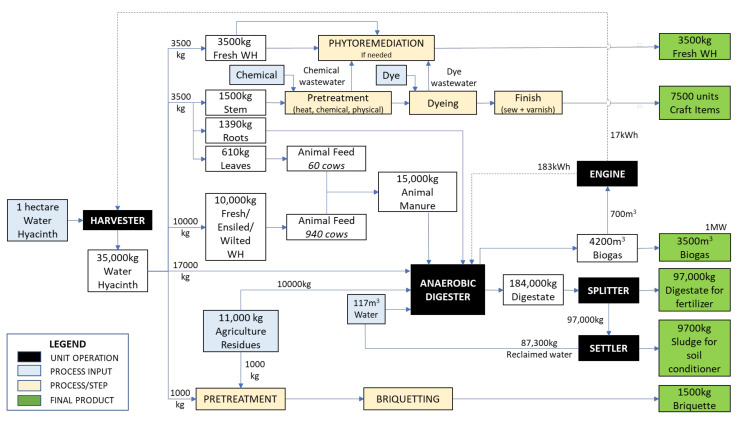
The proposed model for the valorisation of water hyacinth into bioproducts, utilising agricultural residues, reclaimed water and technologies that can be readily adopted by rural communities.

As reviewed in the previous sections, various approaches can be followed to develop useful products from water hyacinth. Any processes can be customised based on the community requirements, resource availability, the skills of the potential workforce and the sustainability of the process. Figure 7 illustrates the proposed model for such an endeavour and includes some general assumptions. It has been reported that water hyacinth coverage may produce 450 tonnes/hectares with a doubling time of seven days [100]. Based on these figures, a harvesting rate of 35 tonnes/hectare/day would ensure that a sufficient amount of the plant remains to sustain the process, yet be plentiful enough to stimulate the rural economy through the generation of valued products, job opportunities and income creation while improving the quality of life. In general, rural communities are socio-economically connected to agriculture. Hence, the proposed model accommodated the labour availability, which is capped at 30% full capacity and eight man-hours for craft production, in which five items per day may be produced from 1 kg of fresh water hyacinth stems. Another assumption concerned the availability of agricultural residues, as it has been reported that up to 20% of total residues can be removed but still maintain yield and soil fertility. As for the farm animals, this model includes cows that consume 10% of their body weight and produce up to 30 kg of manure daily. 

In summary, from one hectare of water hyacinth and the agricultural residues from one plantation hectare, 3500 m^3^ of biogas can be produced through anaerobic digestion, which can be used to provide energy for the community, reducing their dependence on the electricity grid. The energy produced can also be used to power a digester and a harvester, allowing the process to be energetically sustainable and potentially reducing the operational costs. The crafts, bio-briquettes and fresh plant biomass can be sold to interested parties, while any wastewater generated from these processes can be treated through phytoremediation without endangering the ecosystem. The digestate from the AD process may also be used by farmers to augment their crops or sold to gain additional income. Meanwhile, water consumption can be reduced by incorporating reclaimed water from the digester output. The harmony of utilising one form of waste as a resource for another process presents an attractive option for communities seeking improvements in their way of life. This process adopts the circular economy philosophy and acts as a nudge towards investing continuous effort in mitigating the issue of water hyacinth infestation. Meanwhile, incentives are created in the form of income generation, job creation, social empowerment and environmental protection. 

Restoring the ecosystem invaded by water hyacinth will involve long-term commitment from communities, utilising their unique resources and current economic activities as suggested by the microeconomic model. As an integrated approach is crucial for a long-term solution for water hyacinth invasion, the model may be used to design systems, plan logistics, predict targeted product yields, and assist communities in developing their own business models. Systematic harvesting of the plant will eventually help in controlling the spread of the invasion, which provides an opportunity for the ecosystem to naturally heal. Less water hyacinth mat cover on water bodies will increase sunlight penetration, reduce turbidity, increase dissolved oxygen levels, and subsequently improve water quality. This will then restore the habitat conditions for aquatic organisms, allowing them to return and flourish. Once a balance has been achieved between humans, water hyacinth and the aquatic ecosystem, a new equilibrium in ecosystem stability can be obtained, one that satisfies the nexus of economic strength, environmental protection, and social empowerment. Therefore, water hyacinth invasion should be treated as an opportunity for both the community and the environment to develop adaptive capacities and build resilience in coping with environmental disturbances and achieving sustainability.

## Data Availability

No new data were created or analysed in this study. Data sharing is not applicable to this article.
